# Association between Prior Malignancy Exclusion Criteria and Age Disparities in Cancer Clinical Trials

**DOI:** 10.3390/cancers14041048

**Published:** 2022-02-18

**Authors:** Roshal R. Patel, Rose Parisi, Vivek Verma, Ramez Kouzy, Joseph Abi Jaoude, Timothy A. Lin, Clifton David Fuller, Noam A. VanderWalde, Reshma Jagsi, Benjamin D. Smith, Beverly Ashleigh Guadagnolo, Charles R. Thomas, Ethan B. Ludmir

**Affiliations:** 1 Department of Radiation Oncology, The University of Texas MD Anderson Cancer Center, Houston, TX 77030, USA; roshal.r.patel@kp.org (R.R.P.); vivek333@gmail.com (V.V.); rkouzy@mdanderson.org (R.K.); jbabi@mdanderson.org (J.A.J.); cdfuller@mdanderson.org (C.D.F.); bsmith3@mdanderson.org (B.D.S.); aguadagn@mdanderson.org (B.A.G.); 2 Department of Internal Medicine, Kaiser Permanente Los Angeles Medical Center, Los Angeles, CA 90027, USA; 3 Albany Medical College, Albany, NY 12208, USA; parisir@amc.edu; 4 Department of Radiation Oncology, The Johns Hopkins University School of Medicine, Baltimore, MD 21205, USA; tlin50@jh.edu; 5 Department of Radiation Oncology, West Cancer Center and Research Institute, Memphis, TN 38138, USA; nvanderw@westclinic.com; 6 Department of Radiation Oncology, University of Michigan, Ann Arbor, MI 48109, USA; rjagsi@med.umich.edu; 7 Department of Radiation Oncology, Oregon Health and Science University, Portland, OR 97239, USA; thomasch@ohsu.edu

**Keywords:** prior malignancy exclusion criteria, age disparities, phase III, cancer clinical trials

## Abstract

**Simple Summary:**

Recent studies have shown that the incidence of age disparities in cancer clinical trials may be increasing over time. Excluding patients with prior malignancies is one such eligibility criterion through which elderly may inadvertently be excluded from clinical trial participation. While strict enrollment criteria may improve internal validity of studies, they can also negatively impact generalizability of results. As such, we sought to characterize the incidence of prior malignancy exclusion criteria in phase III cancer clinical trials and assess if this eligibility criterion may directly contribute to age disparities. These data support efforts to modernize eligibility criteria and inform best practices regarding acceptable versus unacceptable exclusionary timeframes for prior malignancy exclusion criteria.

**Abstract:**

Prior malignancy exclusion criteria (PMEC) are often utilized in cancer clinical trials; however, the incidence of PMEC and the association of PMEC with trial participant age disparities remain poorly understood. This study aimed to identify age disparities in oncologic randomized clinical trials as a result of PMEC. Using a comprehensive collection of modern phase III cancer clinical trials obtained via ClinicalTrials.gov, we assessed the incidence and covariates associated with trials excluding patients with prior cancers within 5+ years from registration (PMEC-5). Using the National Cancer Institute Surveillance, Epidemiology, and End Results (SEER) database, we further sought to determine the correlation between PMEC-5 and age disparities. PMEC-5 were used in 41% of all trials, with higher PMEC-5 utilization among industry-supported trials as well as trials evaluating a targeted therapy. Comparing trial patient median ages with population-matched median ages by disease site and time-period, we assessed the association between PMEC-5 and age disparities among trial participants. PMEC-5 were independently associated with heightened age disparities, which further worsened with longer exclusionary timeframes. Together, PMEC likely contribute to age disparities, suggesting that eligibility criteria modernization through narrower PMEC timeframes may work toward reducing such disparities in cancer clinical trial enrollment.

## 1. Introduction

Second malignancies account for over a sixth of all reported cancers [1]. Cancer clinical trials often utilize eligibility criteria that exclude patients who have had a previous cancer based on the possibility that overlapping natural history of antecedent and new tumors may confound the validity of endpoints (i.e., survival) to be studied [2]. Prior malignancy exclusion criteria (PMEC) in oncology clinical trials have come under scrutiny due to concerns regarding generalizability of results [3,4,5].

Cancer clinical trials may demonstrate improved outcomes compared with non-trial studies due to enrollment of patients with fewer comorbidities as a result of stricter eligibility criteria [6]. Although such a clinical trials effect is controversial [7], stricter eligibility criteria may also increase the potential for incomplete accrual through up-front exclusion of otherwise-eligible patients [8]. Notably, older patients are known to have lower rates of participation in cancer clinical trials [9,10,11]. Previous studies have identified age-based exclusion criteria [12,13]; however, the extent to which age disparities exist due to prior malignancy criteria has not been identified. 

In the context of known age disparities among trial participants, we hypothesized that PMEC, which may disproportionately impact older patients, would be associated with age disparities. Therefore, we sought to assess the incidence and correlates of PMEC in cancer clinical trials, specifically trials that excluded patients with prior malignancies within five years or more from the time of registration (PMEC-5); we further sought to determine whether PMEC utilization is associated with age disparities among trial participants. Using these data our goal was to inform clinical trial practices in a way that may reduce age disparities and limitations in clinical trial enrollment. 

## 2. Materials and Methods

### 2.1. Inclusion Criteria and Data Analysis

**Figure 1:**
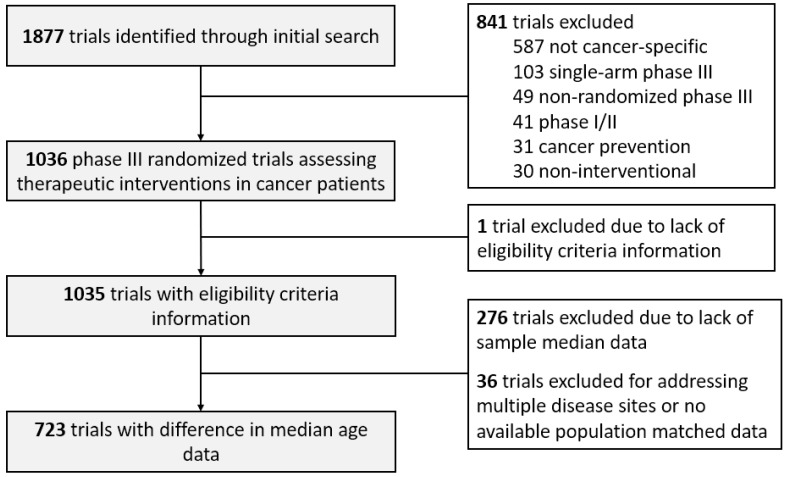
Trial selection criteria for PMEC and DMA analyses.

We identified eligible studies by querying ClinicalTrials.gov (access on 20 February 2020) with the search criteria: terms: “cancer”; study type: “all studies”; status: excluded “not yet recruiting”; phase: “phase 3”; and study results: “with results”. Of 1877 screen-identified trials, 1036 were eligible as cancer-specific phase III randomized trials addressing a therapeutic intervention enrolling between 1991 and 2020 [9]. PMEC were defined as general exclusions for prior cancers, even if certain exceptions for specific malignancies were made (primarily non-melanomatous skin cancers). Trials that excluded active malignancies only or those that made exceptions for stage I/II cancers in complete remission or treated cancers with low risk for metastases were not considered to have PMEC. In evaluating age disparities, we calculated the difference in median age (DMA) by subtracting the median participant age for each trial from the median age for the respective disease site acquired from the National Cancer Institute Surveillance, Epidemiology, and End Results (SEER) database [9]. Each trial was compared with the SEER database median age during the period of trial enrollment. Trials addressing multiple sites or those lacking median age data were excluded from DMA analysis (*n* = 312, Figure 1). Trials with missing data were excluded from analysis. 

### 2.2. Statistical Analysis

Pearson’s Chi-squared tests and binary logistic regression analyses of trial factors associated with PMEC-5 were conducted using SPSS Statistics, version 26 (IBM Corp., Armonk, NY, USA). Mann–Whitney *U* tests and Kruskal–Wallis tests were used to assess age disparities across covariates. Multivariable analysis was conducted via multiple linear regression; variables included in the model were those significant (*p* < 0.05) on univariable analysis. 

## 3. Results

### 3.1. Incidence of Prior Malignancy Exclusion Criteria 

Of 1036 phase III cancer clinical trials (total enrollment of 663,026 patients), 421 trials (41%) had no PMEC; those with PMEC excluded prior cancers within timeframes of 1 year (8 trials, 1%), 2 years (43, 7%), 3 years (135, 22%), 5 years (348, 57%), or 10 years/indefinitely (80, 13%). One trial was excluded from this analysis due to lack of eligibility criteria information. PMEC-5 included all trials that excluded patients if they had prior malignancies within 5 years, 10 years, or indefinitely prior to the time of enrollment; therefore, 429 trials (of 1035, 41%) had PMEC-5. Table 1 highlights trial-related factors and outcomes associated with PMEC-5. Higher rates of PMEC-5 utilization were identified among industry-funded trials (44% vs. 33%, *p =* 0.002), systemic therapy trials (48% vs. 20%, *p* < 0.001), and targeted therapy trials (46% vs. 36%, *p* = 0.001). By disease site, melanoma trials had the highest rate of PMEC-5 utilization (55%), and hematologic trials had the lowest (30%, *p* < 0.001). Trials with PMEC-5 were similarly likely to meet their primary endpoint (*p =* 0.78) and accrue to completion (*p =* 0.13). 

### 3.2. Prior Malignancy Exclusion Criteria and Age Disparities

Next, we sought to assess whether PMEC-5 utilization was associated with age disparities. The mean DMA across all evaluable trials was −5.8 years (standard error 0.23 years). Table 2 displays factors statistically significantly associated with age disparities. Trials with PMEC-5 were more likely to demonstrate age disparities (mean DMA −6.4 vs. −5.3 years, *p =* 0.001). Targeted therapy (*p =* 0.019), systemic therapy (*p =* 0.028), breast (*p =* 0.012), gastrointestinal (*p* < 0.001), and thoracic (*p* < 0.001) trials were also associated with age disparities. Conversely, radiotherapy (*p =* 0.029), genitourinary (*p* < 0.001), head and neck (*p =* 0.003), and other (*p* < 0.001) trials were associated with fewer age disparities. On multivariable regression modeling, PMEC-5 (*p =* 0.005) remained independently associated with age disparities, along with genitourinary, head and neck, thoracic, and other trials. 

We then examined whether longer exclusionary timeframes demonstrated wider age disparities by focusing on those trials with PMEC exclusionary windows of at least 10 years or indefinitely (PMEC-10). We hypothesized that these trials with stricter PMEC criteria would have further heightened age disparities. The mean DMA for these trials with PMEC-10 was −8.5 years as compared with −5.5 years in those without (*p* < 0.001, Table S1). PMEC-10 remained an independent predictor of age disparities (*p* < 0.001) on multivariable regression, along with trials of specific disease sites.

## 4. Discussion

These data demonstrate pervasive utilization of PMEC across phase III cancer clinical trials. Furthermore, there is an independent association between PMEC and age disparities among clinical trial participants, an association that also scales with longer timeframes for PMEC. With one-fifth of patients over 65 years having prior malignancies, and the incidence of cancer in older patients (>65) projected to increase by 67% over the next decade, this represents a substantial proportion of patients potentially excluded from clinical trials due to PMEC [5,14]. Older patients are already known to be underrepresented in clinical trials [9,10,11]. While decreased enrollment may be attributed to a combination of factors including that older patients need more social supports and resources, PMEC appear to constitute a consistent deterrent to including older patients [11].

Notably, the impact of prior malignancies on outcomes has been examined across several cancer types including glioblastoma, colorectal, lung, pancreatic, and uterine cancer [5,15,16,17,18,19] The incidence of older patients with prior malignancies ranged from 7% to 25% across these disease sites, generally without differences in cancer-specific survival, irrespective of whether patients had prior malignancy diagnoses [5,15,16,17,18,19,20]. Only one of the aforementioned studies, on colorectal cancer (CRC) [16], noted poorer overall survival (OS) among patients with non-leukemic prior cancers (although CRC-specific survival was improved in these patients); the remaining studies showed no OS difference based on patients’ prior malignancy status [5,15,17,18]. These data suggest that inclusion of patients with prior malignancies may not impact disease-related outcomes, including survival. Nearly half of the trials in our database with a primary endpoint of OS employed PMEC-5. Exploratory analyses within trials that did not employ PMEC may allow for comparison of outcomes between enrolled patients with and without prior malignancy diagnoses. This could help further shed light on the potential impact of prior malignancies on clinical outcomes and could assist in guiding evidence-based recommendations for future trial eligibility criteria. 

In recent years, the American Society of Clinical Oncology and Friends of Cancer Research joint working groups have discussed broadening and modernizing eligibility criteria in cancer clinical trials to improve participation, access to investigational therapies, and generalizability of results [4]. Their guidance suggests that oncologic clinical trials include patients with previously treated malignancies older than 2 years [4,21]. These guidelines further suggest that patients with concurrent malignancies should be included if they are clinically stable and do not require tumor-directed treatment for their concurrent malignancy [4]. Although evidence is limited with respect to these recommendations, our study provides quantitative data to assist in drafting evidence-based eligibility criteria. Considering our data highlighting PMEC association with age disparities over specific PMEC time-windows, we recommend trialists consider using exclusion criteria based on an estimated risk of prior malignancy relapse over a specified follow-up period (for example, exclusion of patients with >25% prior malignancy relapse risk over 5 years). This proposed risk-based PMEC model allows such exclusion criteria to be tailored to individual trial- and patient-level considerations.

There are several limitations to this analysis. First, although most trials report comprehensive eligibility criteria on ClinicalTrials.gov, a handful do not. While eligibility criteria for every trial was cross-referenced with available publications and protocols, gaps may exist, and therefore this study may underrepresent rates of PMEC. Second, PMEC trials often made exceptions for certain previous malignancies, most commonly cutaneous basal cell carcinoma (87%), cutaneous squamous cell carcinoma (77%), and cervical carcinoma in situ (71%). Heterogeneity in PMEC exceptions across trials may limit aspects of this analysis. Third, the age disparities analyses utilized domestic SEER data, while many trials enroll patients multi-nationally. Differences in demographics worldwide may limit the applicability of SEER data in this context.

## 5. Conclusions

PMEC are widely utilized among cancer clinical trials. PMEC-5 were used in over 41% of phase III clinical trials over the past three decades, and they are associated with age disparities. These disparities worsen with stricter exclusionary timeframes, which may have implications on older patient enrollment as well as on the generalizability of trial results.

## Figures and Tables

**Table 1:** Factors associated with PMEC-5.

Trial/Author Characteristics		Number of Trials with PMEC-5/Total	Percentage	*p*-Value *
All		429/1035	41.4 (%)	-
Industry funding of trial	Yes	339/765	44.3%	**0.002**
	No	90/270	33.3%	
Cooperative Group Trial	Yes	124/302	41.1%	0.87
	No	305/733	41.6%	
Enrollment Start Year	1991–2000	21/40	52.5%	0.53
	2001–2005	81/190	42.6%	
	2006–2010	165/418	39.5%	
	2011–2015	116/269	43.1%	
	2016–2020	46/118	39.0%	
Disease Site ^ǂ^	Breast	97/181	53.6%	**<0.001**
	Gastrointestinal	52/120	43.3%	
	Genitourinary	65/126	51.6%	
	Head and Neck	14/43	32.6%	
	Skin	18/33	54.5%	
	Thoracic	69/148	46.6%	
	Hematologic	61/207	29.5%	
	Other	53/177	29.9%	
Treatment modality ^§^	Systemic therapy	383/801	47.8%	**<0.001**
	Radiotherapy	9/30	30.0%	
	Surgery	3/9	33.3%	
	Supportive Care	34/195	17.4%	
Targeted Therapy	Yes	257/554	46.4%	**0.001**
	No	172/481	35.8%	
Completed planned accrual ^#^	Yes	215/480	44.8%	0.13
	No	86/222	38.7%	
Trial success (PEP met) ^	Yes	178/400	45.5%	0.78
	No	167/367	44.5%	

Abbreviations: PMEC, prior malignancy exclusion criteria; PEP, primary endpoint; OS, overall survival. * *p*-value reflects Pearson’s Chi-squared tests for all except by Enrollment Start Year (binary logistic regression analysis by year). ^ǂ^ Limited to trials with a defined single disease site. “Other” includes trials of other single disease sites as well as multiple disease sites. ^§^ Primary intervention as part of the randomization. Systemic therapy includes cytotoxic chemotherapy, targeted systemic agents, and similar, with primary endpoint aimed at improved disease-related outcomes. Supportive care trials aimed to reduce disease- or treatment-related toxicity. ^#^ 333 trials did not have final accrual data, in some cases due to trial ongoing. ^ 9 trials were excluded from trial success analysis of whether the PEP was met, as these trials had multiple PEPs with mixed results at the time of publication; 259 trials were excluded for not having any associated publication.

**Table 2:** Factors associated with age disparities—PMEC5.

Trial/Author Characteristics		Number of Trials	Mean DMA (SE), Years	Univariable *p*-Value *	Multivariable Regression *p*-Value
All		723	−5.82 (0.23)	-	-
PMEC-5	Yes	347	−6.36 (0.33)	**0.005**	**0.001**
	No	376	−5.32 (0.32)	-	-
Industry funding of trial	Yes	585	−5.87 (0.26)	0.25	-
	No	138	−5.58 (0.53)	-	-
Cooperative Group Trial	Yes	182	−5.97 (0.46)	0.92	-
	No	541	−5.77 (0.27)	-	-
Enrollment Start Year	1991–2000	30	−4.19 (0.97)	0.64	-
	2001–2005	133	−6.62 (0.52)	-	-
	2006–2010	315	−5.70 (0.37)	-	-
	2011–2015	191	−5.79 (0.45)	-	-
	2016–2020	54	−5.57 (0.75)	-	-
Disease Site ^ǂ^	Breast	142	−7.05 (0.46)	**0.012**	0.68
	Gastrointestinal	97	−7.97 (0.45)	**<0.001**	0.053
	Genitourinary	98	−0.45 (0.57)	**<0.001**	**<0.001**
	Head and Neck	30	−2.46 (1.06)	**0.003**	**<0.001**
	Skin	29	−5.74 (0.82)	0.97	-
	Thoracic	126	−9.05 (0.32)	**<0.001**	**<0.001**
	Hematologic	144	−6.62 (0.59)	0.57	-
	Other	57	−0.98 (0.86)	**<0.001**	**<0.001**
Treatment modality ^§^	Systemic therapy	624	−5.97 (0.25)	**0.028**	0.43
	Radiotherapy	15	−2.92 (1.03)	**0.029**	0.24
	Surgery	5	−10.44 (4.48)	0.45	-
	Supportive Care	79	−4.92 (0.68)	0.11	-
Targeted Therapy	Yes	454	−6.18 (0.29)	**0.019**	0.53
	No	269	−5.19 (0.39)	-	-
				
Completed planned accrual ^#^	Yes	417	−5.48 (0.31)	0.08	-
	No	154	−6.61 (0.52)	-	-
Trial success (PEP met) ^	Yes	344	−5.63 (0.36)	0.65	-
	No	299	−5.90 (0.34)	-	-

Abbreviations: PMEC-5, prior malignancy exclusion criteria for prior cancers within 5 years, 10 years, or indefinitely. * *p*-value reflects Mann–Whitney *U* test, except for Disease Site and Treatment Modality (for which the Kruskal–Wallis test was used) and Enrollment Start Year (for which a linear regression was conducted). ^ǂ^ Limited to trials with a defined single disease site. “Other” includes trials of other single disease sites. Trials with multiple disease sites were excluded. ^§^ Primary intervention as part of the randomization. Systemic therapy includes cytotoxic chemotherapy, targeted systemic agents, and similar, with primary endpoint aimed at improved disease-related outcomes. Supportive care trials aimed to reduce disease- or treatment-related toxicity. ^#^ 152 trials did not have final accrual data, in some cases due to trial ongoing. ^ 100 trials either had multiple PEPs with mixed results or did not have any associated publication.

## Data Availability

All data are publicly available on ClinicalTrials.gov and the National Cancer Institute Surveillance, Epidemiology, and End Results (SEER) database. Datasets used for the analysis can be made available by authors upon reasonable request.

## Supplementary Materials

The following supporting information can be downloaded at: https://www.mdpi.com/article/10.3390/cancers14041048/s1, Table S1: Factors Associated with Age Disparities–PMEC10.

## Abbreviations

CRC: colorectal cancer.; DMA, difference in median age; OS, overall survival; PMEC, prior malignancy exclusion criteria; PMEC-5, prior malignancy exclusion criteria for malignancies within the past 5 years; PMEC-10, prior malignancy exclusion criteria for malignancies within the past 10 years; SEER, Surveillance, Epidemiology, and End Results.
