# Supplementary material for: Association between Prior Malignancy Exclusion Criteria and Age Disparities in Cancer Clinical Trials
Source: Cancers (Basel). 2022 Feb 18;14(4):1048. doi: 10.3390/cancers14041048 (PMC8870379; doi:10.3390/cancers14041048)
Supplement: Supplementary file 1 [file cancers-14-01048-s001.zip › cancers-1570738-supplementary.pdf]

Supplemental Table S1. Factors Associated with Age Disparities – PMEC10.

Trial/Author Characteristics		Number of trials	Mean DMA (SE), years	Univariable p-value*	Multivariable regression p-value
All		723	-5.82 (0.23)	-	-
PMEC-10	Yes	67	-8.52 (0.84)	**<0.001**	**<0.001**
	No	656	-5.54 (0.24)	-	
Industry funding of trial	Yes	585	-5.87 (0.26)	0.25	-
	No	138	-5.58 (0.53)	-	-
Cooperative Group Trial	Yes	182	-5.97 (0.46)	0.92	-
	No	541	-5.77 (0.27)	-	-
Enrollment Start Year	1991-2000	30	-4.19 (0.97)	0.64	-
	2001-2005	133	-6.62 (0.52)	-	-
	2006-2010	315	-5.70 (0.37)	-	-
	2011-2015	191	-5.79 (0.45)	-	-
	2016-2020	54	-5.57 (0.75)	-	-
Disease Site[ǂ]	Breast	142	-7.05 (0.46)	**0.012**	0.56
	Gastrointestinal	97	-7.97 (0.45)	**<0.001**	**0.043**
	Genitourinary	98	-0.45 (0.57)	**<0.001**	**<0.001**
	Head & Neck	30	-2.46 (1.06)	**0.003**	**<0.001**
	Skin	29	-5.74 (0.82)	0.97	-
	Thoracic	126	-9.05 (0.32)	**<0.001**	**<0.001**
	Hematologic	144	-6.62 (0.59)	0.57	-
	Other	57	-0.98 (0.86)	**<0.001**	**<0.001**
Treatment modality[§]	Systemic therapy	624	-5.97 (0.25)	**0.028**	0.72
	Radiotherapy	15	-2.92 (1.03)	**0.029**	0.28
	Surgery	5	-10.44 (4.48)	0.45	-
	Supportive Care	79	-4.92 (0.68)	0.11	-
Targeted Therapy	Yes	454	-6.18 (0.29)	**0.019**	0.32
	No	269	-5.19 (0.39)	-	-
Completed planned accrual[#]	Yes	417	-5.48 (0.31)	0.08	-
	No	154	-6.61 (0.52)	-	-
Trial success (PEP met)[^]	Yes	344	-5.63 (0.36)	0.65	-
	No	299	-5.90 (0.34)	-	-

Abbreviations: PMEC-10, prior malignancy exclusion criteria for prior cancers within 10 years/indefinitely.
*P-value reflects Mann-Whitney *U*-test, except for Disease Site and Treatment Modality (for which the Kruskal-Wallis test was used) and Enrollment Start Year (for which a linear regression was conducted).
[‡]Limited to trials with a defined single disease site. "Other" includes trials of other single disease sites. Trials with multiple disease sites were excluded.
[§]Primary intervention as part of the randomization. Systemic therapy includes cytotoxic chemotherapy, targeted systemic agents, and similar, with primary endpoint aimed at improved disease-related outcomes. Supportive care trials aimed to reduce disease- or treatment-related toxicity.
[#]152 trials did not have final accrual data, in some cases due to trial ongoing.
[^]100 trials either had multiple PEPs with mixed results or did not have any associated publication
